# Fluoroquinolone resistance mechanisms in an *Escherichia coli* isolate, HUE1, without quinolone resistance-determining region mutations

**DOI:** 10.3389/fmicb.2013.00125

**Published:** 2013-05-24

**Authors:** Toyotaka Sato, Shin-ichi Yokota, Ikuo Uchida, Torahiko Okubo, Masaru Usui, Masahiro Kusumoto, Masato Akiba, Nobuhiro Fujii, Yutaka Tamura

**Affiliations:** ^1^Laboratory of Food Microbiology and Food Safety, Department of Health and Environmental Sciences, School of Veterinary Medicine, Rakuno Gakuen UniversityEbetsu, Japan; ^2^Department of Microbiology, Sapporo Medical University School of MedicineSapporo, Japan; ^3^Dairy Hygiene Research Division, Hokkaido Research Station, National Institute of Animal HealthSapporo, Japan; ^4^Bacterial and Parasitic Disease Research Division, Safety Research Team, National Institute of Animal HealthIbaraki, Japan

**Keywords:** AcrAB, efflux pump, *Escherichia coli*, fluoroquinolone resistance, *oqxAB*, *qnrS*

## Abstract

Fluoroquinolone resistance can cause major clinical problems. Here, we investigated fluoroquinolone resistance mechanisms in a clinical *Escherichia coli* isolate, HUE1, which had no mutations quinolone resistance-determining regions (QRDRs) of DNA gyrase and topoisomerase IV. HUE1 demonstrated MICs that exceeded the breakpoints for ciprofloxacin, levofloxacin, and norfloxacin. HUE1 harbored *oqxAB* and *qnrS1* on distinct plasmids. In addition, it exhibited lower intracellular ciprofloxacin concentrations and higher mRNA expression levels of efflux pumps and their global activators than did reference strains. The genes encoding AcrR (local AcrAB repressor) and MarR (MarA repressor) were disrupted by insertion of the transposon IS*3*-IS*629* and a frameshift mutation, respectively. A series of mutants derived from HUE1 were obtained by plasmid curing and gene knockout using homologous recombination. Compared to the MICs of the parent strain HUE1, the fluoroquinolone MICs of these mutants indicated that *qnrS1*, *oqxAB*, *acrAB*, *acrF*, *acrD*, *mdtK*, *mdfA*, and *tolC* contributed to the reduced susceptibility to fluoroquinolone in HUE1. Therefore, fluoroquinolone resistance in HUE1 is caused by concomitant acquisition of QnrS1 and OqxAB and overexpression of AcrAB–TolC and other chromosome-encoded efflux pumps. Thus, we have demonstrated that QRDR mutations are not absolutely necessary for acquiring fluoroquinolone resistance in *E. coli*.

## Introduction

Fluoroquinolones are widely used in the clinical treatment of various bacterial infections, such as urinary tract and blood stream infections caused by *Escherichia coli*. Many studies have reported the isolation of fluoroquinolone-resistant strains (Peña et al., 1995; Cizman et al., 2001; Sanchez et al., 2012). Fluoroquinolone resistance is mainly caused by point mutations in the quinolone resistance-determining regions (QRDRs) of the DNA gyrase (encoded by *gyrA* and *gyrB*) and topoisomerase IV (encoded by *parC* and *parE*) subunits (Yoshida et al., 1991; Conrad et al., 1996; Heisig, 1996; Breines et al., 1997). A slight decrease in susceptibility to fluoroquinolones is attributed to a single mutation in *gyrA*. Secondary mutations in *gyrA* and additional mutations in *parC* and/or *parE* are required to exceed the breakpoint of the fluoroquinolone MIC (Conrad et al., 1996; Heisig, 1996; Breines et al., 1997).

Recently, we reported a fluoroquinolone-resistant *E. coli* isolate without QRDR mutations, named HUE1 (Sato et al., 2011). Its MICs for fluoroquinolones, such as ciprofloxacin (CIP) and levofloxacin (LVX), exceeded the breakpoints established by the Clinical and Laboratory Standards Institute (CLSI) (Clinical and Laboratory Standards Institute, 2011). HUE1 possesses two plasmid-mediated quinolone-resistant determinants (PMQRs), viz., *oqxAB* and *qnrS*. In this bacterium, OqxAB is a plasmid-encoded efflux pump; however, the gene is present on the chromosomal DNA in most *Klebsiella pneumoniae* and *Enterobacter cloacae* strains (Bin Kim et al., 2009). The presence of this pump confers resistance to several antimicrobial agents, such as olaquindox (OLA), trimethoprim (TMP), and chloramphenicol (CHL), and decreases bacterial susceptibility to fluoroquinolones (Hansen et al., 2007). QnrS, on the other hand, is a member of the pentapeptide-repeat protein family that protects DNA gyrase (and probably also topoisomerase IV) from binding to fluoroquinolones, thereby decreasing fluoroquinolone susceptibility (Jacoby, 2005). However, acquisition of these PMQRs alone results in only a low level of fluoroquinolone resistance, with MICs that do not exceed the breakpoints for fluoroquinolones (Jacoby, 2005; Hansen et al., 2007). *E. coli* isolates lacking QRDR mutations in *gyrA* and *parC* and showing concomitant acquisition of *oqxAB* and *qnrS* have previously been reported in China; however, these isolates did not exceed the breakpoint for CIP (Zhao et al., 2010).

Our previous findings suggested that the fluoroquinolone resistance of HUE1, which lacks QRDR mutations, is associated with not only with the presence of *oqxAB* and *qnrS* but also with other fluoroquinolone-resistance mechanism(s) (Sato et al., 2011). In the current study, we investigated the fluoroquinolone-resistance mechanisms of the HUE1 strain.

## Methods

### Bacterial isolates

*E. coli* HUE1 had been isolated from the urinary catheter of a 77-year-old female patient at Hokkaido University Hospital (Sapporo, Japan) in 2007 (Sato et al., 2011). The somatic (O) serotype was determined by the slide agglutination test by using *Escherichia coli* O antisera (Denka Seiken, Tokyo, Japan), and the flagellar (H) serotype was determined using reference sera obtained from the Statens Serum Institut (Hillerød, Denmark).

### Susceptibility testing and genetic analysis

Norfloxacin (NOR) was purchased from Sigma-Aldrich (St Louis, MO). Other antibiotics were obtained as described previously (Sato et al., 2011). Susceptibility to fluoroquinolones [CIP, LVX, urifloxacin (URX), sitafloxacin (STX), and NOR], nalidixic acid (NAL), CHL, and TMP was determined by the agar plate dilution method, according to CLSI guidelines (Clinical and Laboratory Standards Institute, 2011). Phe-Arg-β-naphthylamide (PAβN; final concentration, 20 mg/L), which is an inhibitor of the resistance-nodulation-division (RND)-type efflux pump, was purchased from Sigma-Aldrich.

The presence of *oqxA*, *oqxB*, and *qnrS* was determined by PCR (Sorensen et al., 2003; Cattoir et al., 2007). Full-length nucleotide sequences of *oqxAB*, *qnrS*, *acrA*, *acrB*, *acrR*, *acrE*, *acrF*, *acrS*, *tolC*, *soxS*, *soxR*, and *rob* were determined by PCR and direct sequencing by using the primer pairs listed in Table 1. The nucleotide sequence of *marR* was determined as previously described (Lindgren et al., 2003). Nucleotide sequences were determined using a BigDye Terminator v3.1 Cycle Sequencing Kit (Life Technologies, Carlsbad, CA), and sequencing was performed in a 3130 Genetic Analyzer (Life Technologies). The *oqxAB* nucleotide sequence of HUE1 was submitted to GenBank (accession number AB601773). All gene sequences, except those of *oqxAB* and *qnrS*, were compared with those of *Escherichia coli* strain K12 substrain MG1655, which was deposited in GenBank (accession number U00096), as the reference strain.

**Table 1:** **Sequences of primers used for PCR and DNA sequencing in this study**.

**Gene**	**Forward primer or fluorescent probe (5′–3′)**	**Reverse primer (5′–3′)**
*acrA*	acrA-1F (caccggcagtttgaggatcg)	acrA-1R (gcgcggatcaatctggctta)
	acrA-2F (gtcgttgctggactgggtca)	acrA-2R (atgaacaaaaacagagggtttacg)
*acrB*	acrB-1F (tcaatgatgatcgacagtatggct)	acrB-1R (ggaacaactggcgagcaaac)
	acrB-2F (agcggaacgaccagcataac)	acrB-2R (gcgggacgtggtcagaatac)
	acrB-3F (ccagcctggtcaatcagctc)	acrB-3R (gcgtgttatggcggaagaag)
	acrB-4F (cgaataccgccgacagtacc)	acrB-4R (ggatgaacccgaatgagctg)
	acrB-5F (caggattttgccgaactcttca)	acrB-5R (ataaccagcaagccgcaagc)
*acrE*	acrE-F(atagccgaagttcgcccaca)	acrE-R (ctgcgggggtatcggtagtg)
*acrF*	acrF-1F (cagtcaggcgattggcgata)	acrF-1R (accaccgagccgtcactgtt)
	acrF-2F (cagcgttaccagggcaacaa)	acrF-2R (attttgccgacgctgttggt)
	acrF-3F (cgctgcttaaacccgtctctg)	acrF-3R (cagtcgcggagagccataca)
	acrF-4F (cgctgggtgggacttacgtt)	acrF-4R (ttatcctttaaagcaacggcgga)
*acrR*	acrR-F (atcagaacgaccgccagagg)	acrR-R (ttattcgttagtggcaggattacga)
*acrS*	acrS-F (ttacatgacacttaattcattcgtttga)	acrS-R (tgcacatcgctgccttcagt)
*oqxAB*	oqxAB-1F (acatttaccggaataaaaat)	oqxAB-1R (ggcgaggttttgatagtgga)
	oqxAB-2F (acggtgtacgtctactttga)	oqxAB-2R (gtctcggcaatcactttcg)
	oqxAB-3F (gcgcgcggagtatcccggcg)	oqxAB-3R (ccgcatccttattgttgagc)
	oqxAB-4F (atcgagatgggttccggtag)	oqxAB-4R (taaacggacggaaaatccag)
	oqxAB-5F (tggcggccctgctgttaaag)	oqxAB-5R (gataggtctgcagcgtaccg)
	oqxAB-6F (ctggacgtgcaggtcgatcg)	oqxAB-6R (gataaaggcgatggaggtcat)
	oqxAB-7F (gagctgtcgaagcagatcct)	oqxAB-7R (tgcgacccggtgccggaaat)
*qnrS*	qnrSseq-F (ttagtcaggataaacaacaataccca)	qnrSseq-R (atggaaacctacaatcatacatatcgg)
*rob*	robA-F (catctggacgcccctgcatt)	robA-R (agccaatggccccagcatta)
*soxSR*	soxSR-F (gcgctattgccagggatggt)	soxSR-R (tgtgttgacgtcgggggaaa)
*tolC*	tolC-1F (cgggcaggttgtctggctta)	tolC-1R (ctggctcaagcgtgcctgta)
	tolC-2F (gctgcgctgaatgtcgaaaa)	tolC-2R (tgcgtggcgtatggattttg)

### Transformation of plasmids derived from HUE1 into DH5α

Plasmids were isolated from HUE1 as described previously (Kado and Liu, 1981). The plasmids were electroporated into *E. coli* DH5α (Takara, Shiga, Japan) by using an ECM600 (BTX, San Diego, CA, USA) under the following conditions: voltage, 1.8 kV; capacitance, 25 μF; and resistance, 200 ohms. *oqxAB* and/or *qnrS* transformants were screened using Muller-Hinton (MH) agar containing CIP, TMP, and CHL at concentrations ranging from 2- to 8-fold of the respective MIC values.

### Plasmid curing, plasmid re-introduction, and southern hybridization

Curing of plasmids and generation of spontaneous mutants were performed as previously described, with slight modifications (Deane and Rawlings, 2004). Briefly, strains were grown in 4 mL LB broth at 40°C and incubated for periods ranging from a week to a month. Then, clones were picked and grown in MH agar containing sub-MIC concentrations of CIP, TMP, and CHL or no antimicrobials, in order to obtain *oqxAB*- and/or *qnrS*-cured clones. Re-introduction of plasmids harboring *oqxAB* and/or *qnrS* into plasmid-cured mutants was performed using electroporation as described above. The presence of *oqxA*, *oqxB*, and *qnrS* was detected by PCR and Southern hybridization of plasmids, as previously described (Tamamura et al., 2011). Probes for *oqxB* and *qnrS1* were prepared by PCR by using specific primers, as described previously (Cattoir et al., 2007; Bin Kim et al., 2009). Probe labeling was carried out using a PCR DIG labeling mix (Roche Diagnostics, Tokyo, Japan).

### Real-time reverse-transcription (RT) PCR

Overnight cultures were diluted 1:100 in LB broth and grown to the mid-logarithmic phase. RNA was isolated using an RNeasy Mini kit (Qiagen, Hilden, Germany). Gene expression was estimated by quantitative real-time reverse-transcription (RT)-PCR by using the probe and primer pairs shown in Table 2. RT-PCR was performed using a QuantiTect Probe RT-PCR kit (Qiagen) in 20 μL reactions containing 2.5 ng of purified RNA, 0.2 μM of probe, and 0.5 μM of each of the forward and reverse primers. The cycling conditions included reverse transcription at 50°C for 20 min and PCR involving initial activation at 95°C for 15 min and 45 cycles each consisting of 1 min at 55°C and 30 s at 60°C, in a LightCycler 480 system (Roche, Mannheim, Germany). The *E. coli* strain AG100 (K-12 *argE3 thi-1 rpsL xyl mtl* D(*gal-uvrB*) *supE44*) (Okusu et al., 1996), which was gifted by Dr. Helen I. Zgurskaya (University of Oklahoma, USA), was used as a control. Expression levels of *gapA* were used to normalize expression ratios. Data, except for those of *oqxB* and *qnrS1*, were calibrated against expression levels in AG100, which were set as 1, to determine fold changes in expression. Data for *oqxB* and *qnrS1* were calibrated to the respective levels in HUE1, which were set as 1. The data shown represent the mean values of three independent experiments.

**Table 2:** **Sequences of real-time RT-PCR probes and primers for RT-PCR or construction of knockout mutants designed in this study**.

**Gene**	**Forward primer or fluorescent probe (5′–3′)**	**Reverse primer (5′–3′)**	**Purpose**
*acrA*	acrART-F (ctatcaccctacgctctatcttc)^a^	acrART-R (gcgcgcacgaacatacc)^a^	RT-PCR
	acrART-P (cgaacccggatcacactct)^a^^,^^b^	-	RT-PCR
	acrARed-F (atgaacaaaaacagagggtttacgcctctggcggtcgttctgatgctaattaaccctcactaaagggcg)	acrARed-R (agacttggactgttcaggctgagcaccgcttgcggcttgctggttattaatacgactcactatagggctc)	Construction of *acrA* knockout mutant
*acrB*	acrBRT-F (gcggtcgtgtgaagaaagttta)	acrBRT-R (actcccaacgagaagaggagaa)	RT-PCR
	acrBRT-P (tgaccatcagcagcacgaacataccagt)^b^	-	RT-PCR
	acrBRed-F (atgcctaatttctttatcgatcgcccgatttttgcgtgggtgatcgcaattaaccctcactaaagggcg)	acrBRed-R (tcaatgatgatcgacagtatggctgtgctcgatatcttcattcttgctaatacgactcactatagggctc)	Construction of *acrB* knockout mutant
*acrD*	acrDRT-F (gcaacgccgaacgctacg)	acrDRT-R (cacggtcttccagcggtaag)	RT-PCR
	acrDRT-P (caggaacaggaacaccatgccgccaa)^b^	-	RT-PCR
	acrDREd-F (atggcgaatttctttattgatcgccccatttttgcctgggtgctggcaataattaaccctcactaaagggcg)	acrDRed-R (ttattccgggcgcggcttcagcgggaagcggcggcgcaccagcacaaagataatacgactcactatagggctc)	Construction of *acrD* knockout mutant
*acrE*	acrERT-F (cctcctgccctcctttattctg)	acrERT-R (aacggtaacctgcggttcac)	RT-PCR
	acrERT-P (ttctcttctcccttatcgttacaaccggcg)^b^	-	RT-PCR
*acrF*	acrFRed-F (atggcaaacttttttattcgacgaccgatatttgcatgggtgctggccataattaaccctcactaaagggcg)	acrFRed-R (ttatcctttaaagcaacggcggatcaccacaaagaacaccggtacgaagataatacgactcactatagggctc)	Construction of *acrF* knockout mutant
*acrR*	T7A1ACRR-F (actt*aagctt*AAAAGAGTATTGACTTAAAGTCTAACTATAGGATACTTACAGCCATAGGAGGacagctatggcacgaaaaaccaaac)^c^	ACRR-R (ttaagcttcttattcgttagtggcagg)	Plasmid construction of wild type-*acrR*
*gapA*	gapART-F (aaaggcgctaacttcgacaa)	gapART-R (gaacggtggtcatcagacct)	RT-PCR
	gapART-P (caacgataacttcggcatca)^b^	-	RT-PCR
*marA*	marART-F (gccgtaagatgacggaaatcg)	marART-R (gaaggttcgggtcagagtttg)	RT-PCR
	marART-P (agagtatcggctcgttactttccttcagct)^b^	-	RT-PCR
*marR*	T7A1MARR-F (actt*aagctt*AAAAGAGTATTGACTTAAAGTCTAACTATAGGATACTTACAGCCATAGGAGGacagctgtgaaaagtaccagcgatc)^c^	MARR-R (ttaagcttcttacggcaggactttcttaagc)	Plasmid construction of wild type-*marR*
*mdfA*	mdfART-F (ccatgtgctgccctggga)	mdfART-R (gtcacgaccgagttctttcag)	RT-PCR
	mdfART-P (ttgccgcattggcagcgatctcctt)^b^	-	RT-PCR
	mdfARed-F (atgcaaaataaattagcttccggtgccaggcttggacgtcaggcgttactaattaaccctcactaaagggcg)	mdfARed-R (ttacccttcgtgagaatttcccatctgtttatcttttaaaaagataaccataatacgactcactatagggctc)	Construction of *mdfA* knockout mutant
*mdtK*	mdtKRT-F (gcctgctggtgaacatccc)	mdtKRT-R (gcaaggaacatgacccaatacac)	RT-PCR
	mdtKRT-P (tagccacgccacaaccaacgccac)^b^	-	RT-PCR
	mdtKRed-F (gtgcagaagtatatcagtgaagcgcgtctgttattagcattagcaatcccaattaaccctcactaaagggcg)	mdtKRed-R (ttagcgggatgctcgttgcagaatgatggctgacggcagacgttgcaggataatacgactcactatagggctc)	Construction of *mdtK* knockout mutant
*ompC*	ompCRT-F (aacggtcgtgacgcactg)	ompCRT-R (cgatgtaagcagcggtgttc)	RT-PCR
	ompCRT-P (acggcgtcggcggttctatcactt)^b^	-	RT-PCR
*ompF*	ompFRT-F (gaagctcaacctcttggcaac)	ompFRT-R (gccgctggtgtttgtaaatttattag)	RT-PCR
	ompFRT-P (cgggtttcaccgtagttcgctgcca)^b^	-	RT-PCR
*oqxAB*	oqxBRT-F (tggtggtgcatctgttctcc)	oqxBRT-R (catccttcactttcagcgtgg)	RT-PCR
	oqxBRT-P (cgcatatacagcgagtcgtacttcccgc)^b^	-	
	oqxABRed-F (atgagcctgcaaaaaacctggggaaacattcacctgaccgcgctcggcgaattaaccctcactaaagggcg)	oqxABRed-R (ctaggcgggcagatcctcctggaccggcttcctgcgggtcaccagtttcctaatacgactcactatagggctc)	Construction of *oqxAB* knockout mutant
*qnrS*	qnrSRT-F (aatcatacatatcggcaccacaac)	qnrSRT-R (agcacgtcgaaagtcgctg)	RT-PCR
	qnrSRT-P (tgatctcaccttcaccgcttgcacattc)^b^	-	RT-PCR
	qnrSRed-F (atggaaacctacaatcatacatatcggcaccacaacttttcacataaaattaaccctcactaaagggcg)	qnrSRed-R (gtcaggataaacaacaatacccagtgcttcgagaatcagttcttgcttaatacgactcactatagggct)	Construction of *qnrS* knockout mutant
*rob*	robART-F (agtcgaagcggtattgcagc)	robART-R (ccaagtggcacttacagagaatg)	RT-PCR
	robART-P (ccagaatcggacgcgcagtcaggc)^b^	-	RT-PCR
*soxS*	soxSRT-F (cgtcaccgtgcggaacat)	soxSRT-R (tgtcccatcagaaaattattcagga)	RT-PCR
	soxSRT-P(cgagcatattgaccagccgcttaacattga)^b^	-	RT-PCR
*tolC*	tolCRT-F (ggtacgttgaacgagcaggatc)	tolCRT-R (ccatcagcaatagcattctgttcc)	RT-PCR
	tolCRT-P (ctggcactgaacaatgcgctgagcaa)^b^	-	RT-PCR
	tolCRed-F (atgaagaaattgctccccattcttatcggcctgagcctttctgggttcagaattaaccctcactaaagggcg)	tolCRed-R (tcagttacggaaagggttatgaccgttactggtggtagtgcgtgcggatgtaatacgactcactatagggctc)	Construction of *tolC* knockout mutant

a Primer was designed from nucleotides 818–884 for acrA (corresponding to amino acids 272–294 in AcrA).

b Fluorescent probe (5′–3′) used in RT-PCR. The underlined sequences are complementary to the kit DNA template, which contains a neomycin/kanamycin- or hygromycin-resistance cassette flanked by FRT recombination sites.

c Capital letters show nucleotide sequences of the A1 T7 promoter and Shine-Dalgarno, while “aagctt” in italics indicates the HindIII restriction site.

### Construction of gene deletion mutants

Gene disruption was performed by Red/ET recombination by using the Quick and Easy Gene Deletion kit (Gene Bridges GmbH, Heidelberg, Germany) according to the manufacturer's protocol. The relevant forward and reverse primers are shown in Table 2. Briefly, target gene-specific minigenes, containing a neomycin/kanamycin- or hygromycin-resistance cassette, were constructed by PCR with primers containing 46 bp of upstream and downstream sequences that contained the start codon or the stop codon (or the last codon before the stop codon) of the target genes, respectively (Table 2). The target genes were replaced with minigenes containing the drug-resistance cassette by using Red/ET recombination. The integration of interrupted genes was verified by PCR and DNA sequencing.

### Plasmid construction

Each of the wild-type *acrR* and wild-type *marR* DNA segments were amplified by PCR using DH5α genomic DNA as a template and primers containing the A1 T7 promoter, consensus Shine-Dalgarno, and *Hind*III restriction sites (Table 2), which were designed according to a previous report (Edgar et al., 2012). These PCR products were cloned into the *Hin*dIII site of pUC19 (wt-*acrR* or wt-*marR*), and the plasmids were then transformed into HUE1.

### Accumulation assays

Intracellular CIP concentrations were assayed using a fluorometric uptake assay (Usui et al., 2009). Forty milligrams of wet cells was treated with CIP (final concentration, 10 mg/L) in the presence or absence of carbonyl cyanide *m*-chlorophenylhydrazone (CCCP, Sigma-Aldrich; final concentration, 150 μM). The fluorescence of CIP was measured at excitation and emission wavelengths of 277 and 445 nm, respectively, by using an RF-5000 fluorescence spectrophotometer (Shimadzu, Kyoto, Japan) (Nakaminami et al., 2010). The data shown represent the mean value ± standard deviation values calculated from at least three independent experiments.

### Statistical analysis

Statistical significance was determined by the Student's *t*-test. Differences among more than three groups were determined using the Mann–Whitney *U*-test. A *P*-value of 0.05 or less was considered statistically significant.

## Results

### Characteristics of HUE1 and genetic analysis of OqxAB and QnrS

**Figure 1:**
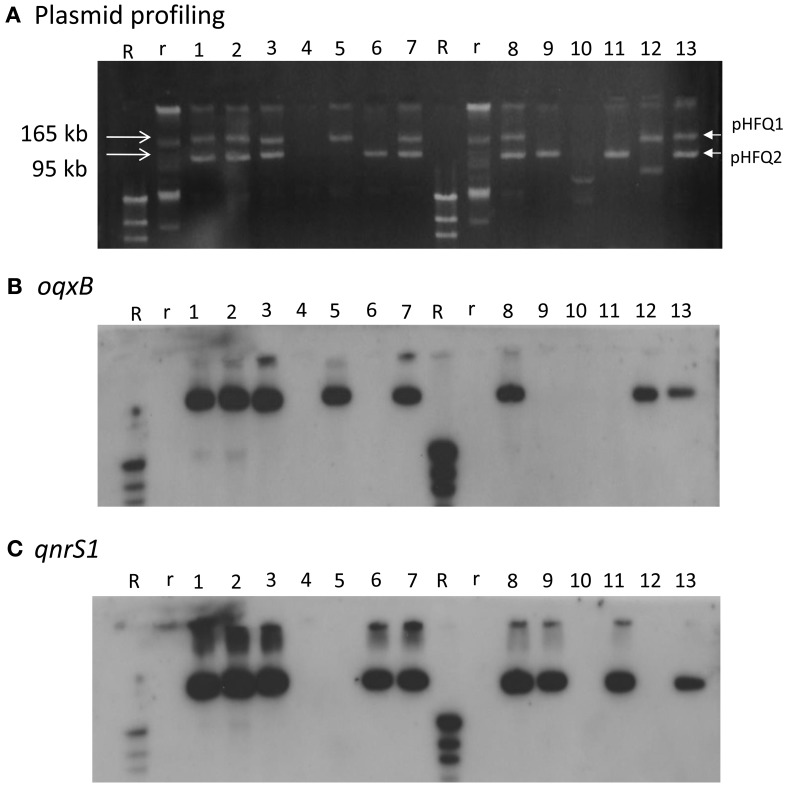
**Plasmid profile and Southern hybridization of *oqxB* and *qnrS1* in HUE1, its *oqxAB*- and/or *qnrS1*-cured strains, and *oqxAB*− and/or *qnrS1*−re-introduced strains. (A)** Plasmid profile. Electrophoresis was performed at 100 V for 70 min with a 0.8% agarose gel. **(B)** Southern hybridization with the *oqxB* probe. **(C)** Southern hybridization with the *qnrS* probe. Lane 1, HUE1; lane 2, HUE1A2; lane 3, HUE1A; lane 4, HUE1A-Curoqxqnr; lane 5, HUE1A-Reoqx; lane 6, HUE1A-Reqnr; lane 7, HUE1A-Reoqxqnr; lane 8, HUE1; lane 9, HUE1B-Curoqx; lane 10, HUE1B-Curoqxqnr; lane 11, HUE1B-Reqnr; lane 12, HUE1B-Reoqx; lane 13, HUE1B-Reoqxqnr; R, DNA Molecular Weight MarkII, DIG-labeled: r, BAC-Tracker Supercoiled DNA Ladder.

HUE1 was identified as ST48 (according to the Max-Planck-Institut für Infektionsbiologie database), phylogenetic group A (Sato et al., 2011), and O125:H37. Its NOR exceeded the breakpoints established by the CLSI, similarly with CIP and LVX previously tested (Sato et al., 2011). A 4421-bp DNA segment containing *oqxAB* and a 647-bp DNA segment containing *qnrS* derived from HUE1 were sequenced. The sequence of *oqxAB* was 100% identical to that of plasmid pOLA52 (accession number EU370913) in an *E. coli* isolate obtained from swine in Sweden (Hansen et al., 2004). The sequence of *qnrS* was 100% identical to that of *qnrS1* in *Shigella flexneri* (accession number AB187515) (Hata et al., 2005). Plasmid profiling and Southern blotting analysis showed that *oqxAB* and *qnrS1* were located on 2 independent plasmids, pHFQ1 (>165 kb) and pHFQ2 (>100 kb), respectively (Figure 1, lane 1).

Introduction of pHFQ1 (carrying *oqxAB*) into DH5α slightly increased the MICs for fluoroquinolones (except for LVX) and NAL, as compared with those for the host strain DH5α (Table 3). Introduction of pHFQ2 (carrying *qnrS1*) resulted in higher MICs for fluoroquinolones and NAL than those seen for DH5α/pHFQ1. However, introduction of both pHFQ1 and pHFQ2 into DH5α did not increase the MICs beyond the breakpoints for fluoroquinolone. Deletion of *oqxAB* or *qnrS* from the transformants by Red/ET recombination reverted MICs for fluoroquinolones to levels similar to those of DH5α.

**Table 3:** **Fluoroquinolones and NAL susceptibilities of HUE1, transformants derived from DH5α, and HUE1mutants derived from plasmid curing and reintroduction**.

**Strain**	**PCR**		**MIC (mg/L)**					
	***oqxAB***	***qnrS1***	**CIP**	**LVX**	**URX**	**STX**	**NOR**	**NAL**
HUE1	+	+	4 (×4)^a^	8 (×16)	4 (×4)	2 (×16)	16	128
**TRANSFORMATION OF pHFQ1 AND pHFQ2**								
DH5α	−	−	0.015	0.03	0.015	0.004	0.03	32
DH5α/pHFQ1	+	−	0.03	0.03	0.03	0.015	0.06	64
DH5α/pHFQ1(Δ*oqxAB*::hyg^r^)	−	−	0.015	0.03	0.015	0.004	0.03	32
DH5α/pHFQ2	−	+	0.125	0.5	0.125	0.125	0.25	128
DH5α/pHFQ2(Δ*qnrS1*::hyg^r^)	−	−	0.015	0.03	0.015	0.004	0.03	32
DH5α/pHFQ1-pHFQ2	+	+	0.25	0.5	0.125	0.25	0.25	>128
**MUTANTS OBTAINED FROM PLASMID CURING**								
<Group A>								
HUE1A and HUE1A2	+	+	2 (×2)	2 (×4)	2 (×2)	1 (×8)	8	64
HUE1A-Curoqxqnr	−	−	0.06 (×2)	0.125 (×4)	0.03 (×1)	0.03 (×4)	0.5	16
HUE1A-Reoqx	+	−	0.125 (×4)	0.125 (×4)	0.125 (×2)	0.06 (×8)	0.5	32
HUE1A-Reqnr	−	+	1 (×2)	1 (×4)	1 (×1)	0.5 (×4)	8	64
HUE1A-Reoqxqnr	+	+	2 (×2)	2 (×4)	2 (×2)	1 (×8)	8	64
<Group B>								
HUE1B-Curoqx	−	+	0.25 (×1)	0.125 (×1)	0.125 (×1)	0.03 (×1)	0.25	4
HUE1B-Curoqxqnr	−	−	0.008 (×1)	0.008 (×1)	0.008 (×1)	0.004 (×1)	0.03	1
HUE1B-Reoqx	+	−	0.125 (×8)	0.06 (×8)	0.03 (×2)	0.03 (×8)	0.25	16
HUE1B-Reqnr	−	+	0.125 (×1)	0.125 (×1)	0.125 (×1)	0.03 (×1)	0.25	4
HUE1B-Reoqxqnr	+	+	1 (×4)	1 (×4)	0.5 (×2)	0.25 (×8)	4	32

a Reduction of MIC by PAβN (-fold),

r resistance.

### Characterization of mutants derived from plasmid curing and plasmid re-introduction

Eleven mutants obtained by curing and re-introduction of the plasmids showed altered fluoroquinolone MICs (Figure 1 and Table 3). These mutants were grouped into two types (groups A and B). In the case of group A, we first obtained two strains, named HUE1A and HUE1A2. Although these two mutants still harbored pHFQ1 (carrying *oqxAB*) and pHFQ2 (carrying *qnrS1*; Figure 1, lanes 2 and 3), the MICs of NAL and fluoroquinolones were 2- or 4-fold lower than those of the parental strain, HUE1. Secondary screening of mutants by using HUE1A yielded a mutant, HUE1A-Curoqxqnr, which had lost pHFQ1 and pHFQ2 (Figure 1, lane 4). This mutant had 16- to 64-fold lower MICs for fluoroquinolones than the parental strain, HUE1A. Re-introduction of pHFQ1 and pHFQ2 into HUE1A-Curoqxqnr yielded HUE1A-Reoqxqnr, in which fluoroquinolone MICs recovered to the levels seen for HUE1A, but not to the MICs of the parent strain, HUE1.

In the case of group B, we first obtained a mutant, HUE1B-Curoqx, which had lost pHFQ1; a second screening using HUE1B-Curoqx yielded HUE1B-Curoqxqnr, which had lost both pHFQ1 and pHFQ2 (Figure 1, lanes 9 and 10). Interestingly, the fluoroquinolone MICs of HUE1B-Curoqxqnr were 512- and 1280-fold lower than those of HUE1 and were 4- to 16-fold lower than those of HUE1A-Curoqxqnr (Table 3).

Similar differences with respect to the MICs of groups A and B were also observed for the mutant series in which pHFQ2 (carrying *qnrS1*) had been re-introduced. However, most fluoroquinolone MICs of group B strains in which pHFQ1 (carrying *oqxAB*) had been re-introduced were only 2- to 4-fold lower than those of mutants in group A.

### Effects of efflux pump inhibitors and genetic analysis of efflux pump components in the HUE1 strain and its mutants

The efflux pump inhibitor PAβN reduced the fluoroquinolone MICs of the HUE1 strain from 4- to 16-fold (Table 3). In mutants derived from plasmid curing in groups A and B, the effects of PAβN were less than in the parental strain, HUE1. Remarkably, the fluoroquinolone MICs of three mutants that lost *oqxAB* in group B (HUE1B-Curoqxqnr, HUE1B-Curoqx, and HUE1B-Reqnr) were barely affected by PAβN.

HUE1 exhibited higher mRNA expression of efflux pump genes (*acrA, acrB, acrE, acrD, mdtK, mdfA*, and *tolC*) and their global activators (*soxS*, *marA*, and *rob*) than the control strains, AG100 and DH5α. In contrast, *ompF* expression was lower in HUE1 than in the control strains (Table 4). Moreover, in HUE1A and HUE1A-Curoqxqnr, the mRNA expression of efflux pumps and their regulatory genes were approximately half of those in HUE1, while those of HUE1B-Curoqxqnr were similar to those in HUE1. The mRNA expression of *qnrS1* and *oqxB* was not significantly different among the HUE1, HUE1A, HUE1A-Reoqxqnr, and HUE1B-Reoqxqnr strains (data not shown).

**Table 4:** **mRNA expression levels of global activators, efflux pumps, and *ompF* genes in HUE1, HUE1A, HUE1A-Curoqxqnr, and HUE1B-Curoqqnr**.

**Strain**	**Expression level (relative amount of AG100; in terms of fold change)**										
	**Global activators**			**Efflux pumps**							**Porin**
	***soxS***	***marA***	***rob***	***acrA***	***acrB***	***acrE***	***acrD***	***mdtK***	***mdfA***	***tolC***	***ompF***
HUE1	9.80	147.77	8.17	4.67	6.81	8.70	8.34	6.43	6.76	2.83	0.19
HUE1A	4.82	80.65	4.56	2.89	3.55	4.42	3.93	3.21	3.70	2.10	0.15
HUE1A-Curoqxqnr	4.99	82.95	4.81	3.04	3.61	4.33	4.06	2.99	3.77	2.21	0.15
HUE1B-Curoqxqnr	10.02	154.69	8.55	4.21	5.42	10.52	10.20	7.66	7.85	3.20	0.24
DH5α	1.83	21.84	1.44	1.28	1.29	0.69	0.59	0.76	1.17	1.62	2.78

**Figure 2:**
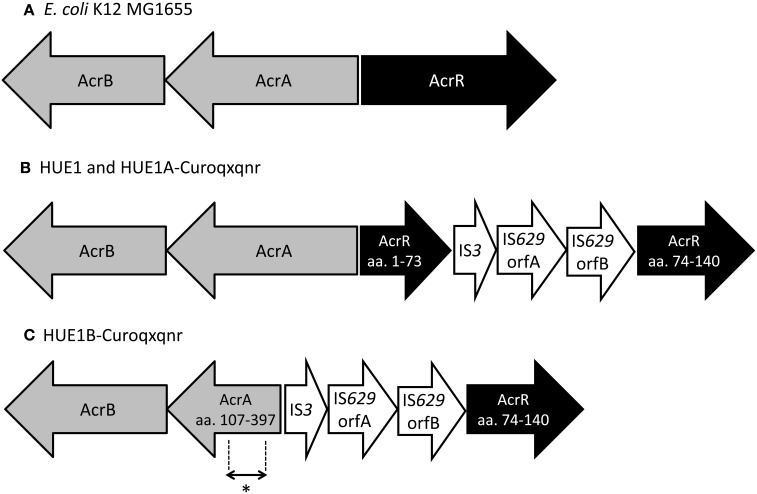
**Schematic representation of the *acrAB*−*acrR* region in HUE1, group A mutants, and group B mutants. (A)** *Escherichia coli* K12 MG1655 (accession no U00096). **(B)** HUE1 and HUE1A-Curoqxqnr (group A). **(C)** HUE1B-Curoqxqnr (group B). aa., Amino acids. ^*^Position of primer pairs (acrART-F and acrART-R) used in RT-PCR (corresponding to amino acids 272–294 in AcrA).

Next, we determined the full DNA sequences of efflux pump genes (*acrA*, *acrB*, *acrE*, *acrF*, and *tolC*) and their regulatory genes (*marR*, *acrS*, *soxS*, *soxR*, *rob*, and *acrR*). Although most genes in HUE1 and its plasmid-cured mutants were wild-type mutants, we found some mutations. *acrR* was disrupted by the insertion of a transposon, an IS*3*–IS*629* element, in HUE1 and group A HUE1A-Curoqxqnr (Figure 2). HUE1B-Curoqxqnr (group B) had a deletion across *acrR* (corresponding to amino acids from Met-1 to Leu-73 of AcrR) and *acrA* (corresponding to amino acids from Met-1 to Asp-106 of AcrA), in addition to the insertion of the IS*3*–IS*629* element. In addition, a nucleotide deletion of cytosine at position 223 of *marR* caused a frameshift in HUE1, HUE1A-Curoqxqnr, and HUE1B-Curoqxqnr.

### Characterization of knockout mutants prepared by Red/ET recombination

We obtained a series of gene knockout mutants by Red/RT recombination. The fluoroquinolone MICs of HUE1-Δ*oqxAB*, HUE1-Δ*qnrS1*, and HUE1-Δ*oqxAB qnrS1* were reduced by 2- or 4-fold, 16- or 32-fold, and 32- or 64-fold, as compared to those of HUE1, respectively (Table 5). HUE1-Δ*acrA*, HUE1-Δ*acrB*, and HUE1-Δ*acrAB* demonstrated 4- or 8-fold lower MICs than HUE1. HUE1-Δ*acrA oqxAB* exhibited 32- or 64-fold lower fluoroquinolone MICs and HUE1-Δ*acrA qnrS1* exhibited 64- or 128-fold lower fluoroquinolone MICs than HUE1. HUE1-Δ*oqxAB* did not exhibit marked changes in fluoroquinolone MICs compared with those of HUE1, while HUE1-Δ*acrAB oqxAB* demonstrated larger fold changes, ranging from 4- to 16-fold lower, than HUE1-Δ*acrAB*.

**Table 5:** **Fluoroquinolones susceptibilities of Red/ET recombination mutants derived from HUE1**.

**Strain**	**MIC (mg/L)**					**MIC reduction (-fold)^a^**
	**CIP**	**LVX**	**URX**	**STX**	**NOR**	
HUE1	4	8	4	2	16	–
HUE1- Δ*oqxAB*	2	2	1	0.5	8	2 or 4
HUE1-Δ*qnrS1*	0.25	0.25	0.125	0.125	0.5	16 or 32
HUE1-Δ*oqxAB qnrS1*	0.06	0.125	0.06	0.06	0.5	32 or 64
HUE1-Δ*acrA*	1	1	0.5	0.25	4	4 or 8
HUE1-Δ*acrA oqxAB*	0.125	0.125	0.125	0.03	0.25	32 or 64
HUE1-Δ*acrA qnrS1*	0.06	0.06	0.03	0.03	0.25	64 or 128
HUE1-Δ*acrB*	1	1	0.5	0.25	4	4 or 8
HUE1-Δ*acrAB*	1	1	0.5	0.25	4	4 or 8
HUE1-Δ*acrAB oqxAB*	0.125	0.125	0.125	0.03	0.25	32 or 64
HUE1-Δ*acrAB qnrS1*	0.06	0.06	0.03	0.03	0.5	32–128,
HUE1-Δ*tolC*	0.06	0.125	0.06	0.015	0.25	64 or 128
HUE1-Δ*tolC acrAB*	0.06	0.125	0.125	0.03	0.25	32 or 64
HUE1-Δ*tolC oqxAB*	0.06	0.125	0.125	0.03	0.25	32 or 64
HUE1-Δ*tolC qnrS1*	0.002	0.004	0.008	0.002	0.015	512–2056
HUE1B-Curoqxqnr	0.008	0.008	0.008	0.004	0.03	512 or 1024
HUE1B-Curoqxqnr-Δ*acrB*	0.008	0.008	0.008	0.004	0.03	512 or 1024
HUE1-Δ*acrF*	2	2	2	1	8	2 or 4
HUE1-Δ*acrD*	2	4	2	1	8	2
HUE1-Δ*mdtK*	2	4	1	1	8	2 or 4
HUE1-Δ*mdfA*	2	4	2	1	8	2 or 4

a Relative value of HUE1.

Knockout of *tolC* markedly decreased fluoroquinolone MICs by 64- or 128-fold, and the MICs of the *tolC* knockout mutant (HUE1-Δ*tolC*) were not altered by additional knockout of *acrAB* or *oqxAB* (HUE1-Δ*tolC acrAB* or HUE1-Δ*tolC oqxAB*)*.* HUE1-Δ*tolC qnrS1* showed the lowest fluoroquinolone MICs, ranging from 512- to 2056-fold lower than those of HUE1. Mutants in which other efflux pump-associated genes, *acrF, acrD, mdtK*, and *mdfA*, were knocked out showed 2- to 4-fold lower fluoroquinolone MICs than did HUE1 (Table 5). The mutant derived from HUE1B-Curoqxqnr with knockout *acrB* (HUE1B-Curoqxqnr-Δ*acrB*) did not show altered fluoroquinolone MICs, when compared to HUE1B-Curoqxqnr.

**Figure 3:**
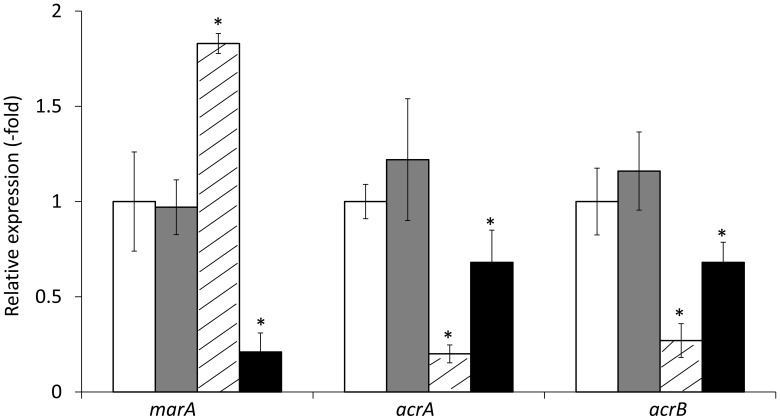
**mRNA expression levels of *marA*, *acrA*, and *acrB* in HUE1 and its *acrR* or *marR* transformants.** The values indicate the expression levels of indicated genes relative to those in HUE1 (fold-changes). White, HUE1; gray, HUE1-pUC19; diagonal line, HUE1-wt-*acrR* (HUE1 strain transformed wild-type of *acrR*); black, HUE1-wt-*marR* (HUE1 strain transformed wild-type *marR*). ^*^p < 0.05 compared to HUE1. Data represent the mean ± standard deviation values calculated from at least three independent experiments.

Transformation of HUE1 with plasmids encoding wild-type *acrR* or wild-type *marR* (HUE1-wt-*acrR* and HUE1-wt-*marR*) resulted in fluoroquinolone MICs that were 2- or 4-fold lower than those for HUE1 (data not shown). HUE1-wt-*acrR* and HUE1-wt-*marR* resulted in reduced mRNA expression levels of *acrA* and *acrB*, and HUE1-wt-*marR* also exhibited a significant reduction in the expression of *marA* compared with that for HUE1 (Figure 3).

### Intracellular fluoroquinolone concentrations

**Figure 4:**
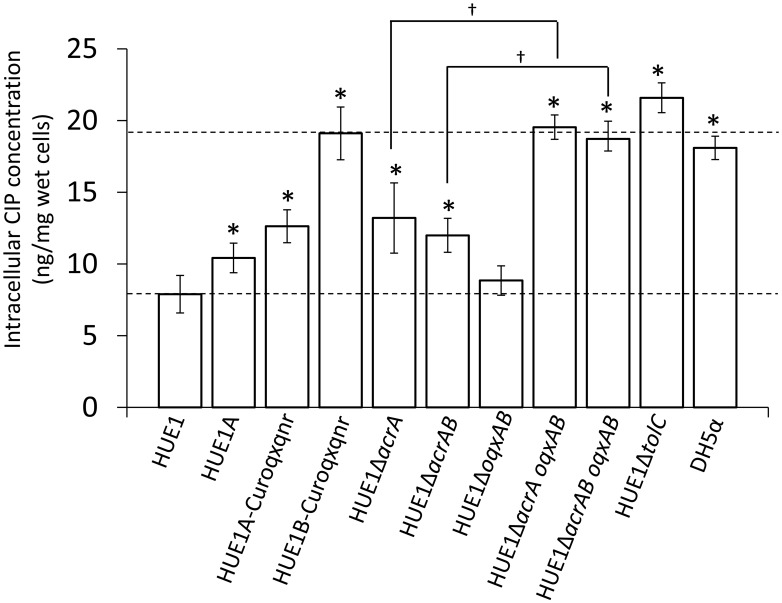
**Intracellular concentrations of CIP in HUE1 and its mutants obtained from plasmid curing and Red/ET recombination.** Data represent the mean ± standard deviation values of the results from three independent experiments. ^*^p < 0.05 compared to HUE1. ^†^*p* < 0.05 between HUE1-Δ*acrA* and HUE1-Δ*acrA oqxAB* and between HUE1-Δ*acrAB* and HUE1-Δ*acrAB oqxAB*, respectively.

The intracellular CIP concentration in HUE1 was approximately 2.3-fold lower than that in DH5α (*p* < 0.05; Figure 4). Intracellular CIP concentrations in HUE1A and HUE1A-Curoqxqnr were minimally, albeit significantly, higher than those of the parental strain, HUE1 (*p* < 0.05). In contrast, the intracellular CIP concentration in HUE1B-Curoqxqnr was markedly higher than those in HUE1, HUE1A, and HUE1A-Curoqxqnr (*p* < 0.05), and was similar to those of HUE1-Δ*acrA oqxAB*, and HUE1-Δ*acrAB oqxAB* (Figure 4). HUE1-Δ*acrA* and HUE1-Δ*acrAB* exhibited significantly increased intracellular CIP concentrations compared with that for HUE1 (*p* < 0.05). However, the intracellular CIP concentrations of *acrD*-, *acrF*-, *mdtK*-, and *mdfA*-knockout HUE1 mutants were slightly higher, but not significantly different from that for HUE1 (*p* > 0.05; data not shown). The intracellular CIP concentration in HUE1-Δ*oqxAB* was also not significantly different from that of HUE1; however, HUE1-Δ*acrA oqxAB* and HUE1-Δ*acrAB oqxAB* exhibited a clear increase in intracellular CIP levels compared to that in HUE1-Δ*acrA* or HUE1-Δ*acrAB* (*p* < 0.05). HUE1-Δ*tolC* showed the highest intracellular CIP concentrations (Figure 4), which were not altered by additional knockout of *acrAB* or *oqxAB* (HUE1-Δ*tolC acrAB* or HUE1-Δ*tolC oqxAB*; data not shown). Addition of CCCP resulted in increased intracellular CIP concentrations in all strains, with levels ranging from 21.8 ± 2.3 to 25.8 ± 5.2 ng/mg wet cells; there were no statistical differences (*p* > 0.05, data not shown).

## Discussion

It is known that QRDR mutations are required for the fluoroquinolone MICs to exceed the breakpoints (Jacoby, 2005; Hansen et al., 2007; Cesaro et al., 2008; Zhao et al., 2010). To our knowledge, a fluoroquinolone-resistant strain without QRDR mutations has been reported only in a laboratory-derived resistant mutant strain of *Acinetobacter baumannii* that had been selected by *in vitro* fluoroquinolone exposure experiments; however, the resistance mechanism of this strain remains unclear (Chopra and Galande, 2011). In the current study, we demonstrated the fluoroquinolone-resistance mechanisms in an *E. coli* clinical isolate, HUE1, which lacks mutations in QRDRs (Sato et al., 2011).

HUE1 possessed two PMQRs on different plasmids, pHFQ1 (harboring *oqxAB*) and pHFQ2 (harboring *qnrS1*). Transformation experiments using DH5α as a host clearly showed that *oqxAB* and *qnrS1* did in fact contribute to fluoroquinolone resistance. However, transformants containing both plasmids still did not exceed the MIC breakpoints.

It has been suggested that some efflux pumps are associated with fluoroquinolone resistance in HUE1. AcrAB-TolC is known to be the most important chromosomally encoded efflux pump related to fluoroquinolone resistance in *Escherichia coli* (Poole, 2005). During plasmid curing, a series of HUE1 group B mutants showed markedly lower fluoroquinolone MICs and higher intracellular CIP concentrations than did HUE1 and its group A mutants, whereas these mutants had mRNA expression levels of efflux pumps similar to those of HUE1. We found that group B mutants possessed deletions across parts of *acrA*. AcrA is an outer membrane protein that binds to AcrB (which plays a role in fluoroquinolone excretion) and TolC (an outer membrane factor) (Kobayashi et al., 2001; Elkins and Nikaido, 2003; Ge et al., 2009). The deleted region of AcrA in group B mutants involves an α−β barrel (53–61 amino acid residues), which forms the AcrB-binding domain, and a part of an α-helical hairpin (99–172 amino acid residues), which forms the TolC-binding domain (Ge et al., 2009). Fluoroquinolone MICs of the Δ*acrA*-knockout HUE1 mutant (HUE1-Δ*acrA*) were similar to those of HUE1B-Reoqxqnr (a group B mutant that possesses *oqxAB* and *qnrS1*), HUE1-Δ*acrB*, and HUE1-Δ*acrAB*. Moreover, the intracellular CIP concentration in HUE1B-Curoqxqnr (a group B mutant that had been cured of *oqxAB* and *qnrS1*) was not different from that in HUE1B-Curoqxqnr-Δ*acrB*. These results suggested that reduction of fluoroquinolone MICs in group B strains was caused by functional disruption of AcrAB–TolC, which did not allow cooperation between AcrA and AcrB and TolC, indicating that fluoroquinolone resistance in HUE1 is partially due to AcrAB–TolC.

AcrAB-TolC expression is controlled by several activators (i.e., *soxS*, *marA*, and *rob*) and repressors (i.e., *acrR*, *marR*, and *soxR*) (White et al., 1997; Poole, 2005; Keeney et al., 2008). HUE1 exhibited higher expression of *acrA*, *acrB*, *tolC* and these activators. In addition, HUE1 had concomitant disruption of the *acrR* and *marR* repressors. AcrR is a local repressor of AcrAB (Wang et al., 2001), and MarR is a repressor of MarA, which is one of the global activators of AcrAB (Keeney et al., 2008). Introduction of wild-type *acrR* or wild-type *marR* to HUE1 actually reduced *acrA* and *acrB* mRNA expression and reduced fluoroquinolone MICs. These data suggested that overexpression of AcrAB–TolC in HUE1 was mediated by the concomitant disruptions of AcrR and MarR. However, other mechanisms are also responsible for the overexpression of AcrAB–TolC and other chromosomally encoded efflux pumps, since the group A series of HUE1 mutants was found to have only approximately half of the mRNA expression levels of chromosomal efflux pump genes, including those encoding AcrAB, despite having the same disruptions in *acrR* and *marR* as HUE1. This will require further investigation.

Other chromosomally encoded efflux pumps, such as AcrEF–TolC and AcrD, belonging to the RND family, MdtK (also known as YdhE or NorE), belonging to the multidrug and toxic compound extrusion family, and MdfA, belonging to the major facilitator superfamily, also excrete fluoroquinolones (Nishino and Yamaguchi, 2001; Poole, 2005; Nishino et al., 2006). The mRNA expression of the related genes were also higher in HUE1, and HUE1 knockout mutants of these genes exhibited reduced fluoroquinolone MICs compared to HUE1, although the influence of these knockouts was weaker than that of AcrAB. These results indicated that the fluoroquinolone-resistance mechanism in HUE1 was most likely associated with the overexpression of AcrAB–TolC, with minor contributions of other chromosomally encoded efflux pumps, in addition to 2 PMQRs, viz., *oqxAB* and *qnrS1*.

OqxAB belongs to the RND-type efflux pump family, as does AcrAB (Hansen et al., 2004). Although OqxAB did not cause marked changes in fluoroquinolone MICs and intracellular CIP concentrations, we observed more distinct changes following *acrAB* deletion. OqxAB requires TolC to excrete AMP, CHL, and OLA, as does AcrAB (Hansen et al., 2004). In this study, knockout of *tolC* markedly decreased fluoroquinolone MICs and intracellular CIP concentrations and negated the effects of both *acrAB* and *oqxAB*. This finding indicated that OqxAB, in conjunction with TolC, was also involved in mediating decreased fluoroquinolone susceptibility. TolC is therefore implicated in the functions of both AcrAB and OqxAB. Although OqxAB minimally contributed to fluoroquinolone resistance in HUE1, it may largely contribute to supplementation of AcrAB functions. TolC is also required for the function of several efflux pumps (other than AcrAB), such as AcrEF and AcrD, in *S. enterica* serovar Typhimurium (Horiyama et al., 2010). Therefore, overexpression of TolC should also be an important element in the fluoroquinolone-resistance mechanism in HUE1 cells, as verified by our finding of the greatest reduction in fluoroquinolone MICs by knockout of *tolC*.

In conclusion, this study is the first to reveal the existence of a fluoroquinolone-resistance mechanism that is mediated without QRDR mutations in the *E. coli* clinical isolate, HUE1. This mechanism involved the concomitant presence of *oqxAB* and *qnrS1* and was associated with the overexpression of AcrAB, other chromosomally encoded efflux pumps, and TolC. HUE1 was identified as phylogenic group A−O125:H37−ST48. This clonal group has also been isolated from humans and animals worldwide (Jorgensen et al., 2010; Bortolaia et al., 2011; Croxall et al., 2011). However, the susceptibilities of ST48 strains to fluoroquinolone compounds vary and have not yet been defined in all cases. Therefore, further epidemiological and molecular biology analyses of the ST48 lineage are required.

### Conflict of interest statement

The authors declare that the research was conducted in the absence of any commercial or financial relationships that could be construed as a potential conflict of interest.
